# Supplementary material for: Glucocorticoid stress hormones stimulate vesicle-free Tau secretion and spreading in the brain
Source: Cell Death Dis. 2024 Jan 18;15(1):73. doi: 10.1038/s41419-024-06458-3 (PMC10796385; doi:10.1038/s41419-024-06458-3)
Supplement: Supplementary file 1 — Supplemental Figures and Figure legends [file 41419_2024_6458_MOESM1_ESM.pdf]

Supplementary Material

Supplemental Figure Legends

Figure S1. Additional control experiments for Figure 1. (**A-B**) Immunoblots and quantification of phospho-GR in hippocampal lysates treated as indicated. Intensity of phospho-GR is expressed as a ratio of total GR for each condition (***$P_{CON\ VS.\ DEX}$ =0.0004, **$P_{DEX\ VS.\ DEX+MIF}$ =0.0023, one-way ANOVA with Tukey's multiple comparisons test). (**C-E**) Representative images (**C**) and quantification (**D-E**) of AT8 (red) and TOMA-1 (green) fluorescence intensity in MAP2 positive (grey) cultured hippocampal neurons treated as indicated. Scale bars, 25 μm. Intensity values are normalized to CON condition (**$P_{CON\ VS.\ DEX}$ =0.0011 for **D**, ****$P_{CON\ VS.\ DEX}$ <0.0001 for **E**, unpaired two-tailed t-test, n=8 fields of view/condition)**.** (**F)** Dot blot assay and quantification of TOMA-1 levels **in EV-**depleted media from hippocampal neurons treated as indicated (**$P_{CON\ VS.\ DEX}$ =0.002, unpaired two-tailed t-test). **(G-K**) Immunoblots and quantification of AT8 and PHF1 levels in hippocampal lysates treated as indicated, expressed as the ratio of tubulin (**H, I**) or total Tau (**J, K**) and normalized to CON condition (**$P_{CON\ VS.\ DEX}$ =0.0012, **$P_{DEX\ VS.\ DEX+MIF}$ =0.004 for **H**, ***$P_{CON\ VS.\ DEX}$ =0.0006, **$P_{DEX\ VS.\ DEX+MIF}$ =0.0031 for **I**, *$P_{CON\ VS.\ DEX}$ =0.0372 for **J**, *$P_{CON\ VS.\ DEX}$ =0.0373 for **K**, one-way ANOVA with Tukey's multiple comparisons test). (**L, M**) Quantification of AT8 or PHF1 levels in EV-depleted media from hippocampal neurons treated as indicated, expressed as ratio of total Tau (see Fig. **1A-D**) (ns, one-way ANOVA with Tukey's multiple comparisons test, n=4 samples/condition). (**N, O**) Quantification of AT8 or PHF1 levels in EV-depleted ACSF from brain slices treated as indicated, expressed as ratio of total Tau (see Fig. **1E-H**) (ns, Tukey's multiple comparisons test). (**P**) Images of EVs on CD9- and CD81-coated capture probes, from hippocampal culture media +/- EV depletion. (**Q-R**) Quantification of particle counts on CD9 (**Q**) and CD81 (**R**) capture probes from the indicated conditions (**$P_{media\ VS.\ EV\ depleted}$=0.0017 for **Q**, ***$P_{media\ VS.\ EV\ depleted}$=0.0002 for **R**, unpaired two-tailed t-test). (**S**) Quantification of ELISA for total Tau levels from media containing cortical or hippocampal brain slices from mice subjected to chronic unpredictable stress (STR) compared to control (CON) conditions (**$P_{CON\ VS.\ STR}$ =0.0079, ***$P_{CON\ VS.\ STR}$ =0.0009, unpaired two-tailed t-test; n=4 samples/condition). (**T**) Quantification of LDH in EV-depleted media from **S**, expressed as ratio of CON (ns, unpaired two-tailed t-test). (**U, V**) Quantification of the change in body weight (last minus first measurement; **U**) and corticosterone levels (zenith/nadir of circadian cycle; **V**) for mice subjected to CON and STR conditions (****$P_{CON\ VS.\ STR}$ <0.0001 for **U**, *$P_{CON\ VS.\ STR}$ =0.0208, unpaired two-tailed t-test; n=16-17 samples/condition). Data is presented as mean ± SEM with

n= 3-4 samples/group (results were confirmed in two independent experiments).

Figure S2. Additional control experiments for Figures 2-3. (A) Quantification of ELISA for total Tau levels in EV-depleted media from the indicated conditions, with values normalized to CON condition (**$P_{CON\ VS.\ DEX}$=0.0013, **$P_{DEX\ VS.\ DEX\ +\ NaClO3}$ =0.0042, one-way ANOVA with Tukey's multiple comparisons test). **(B)** Quantification of ELISA for total Tau levels in EV-depleted ACSF from the indicated conditions, with values normalized to CON condition (**$P_{CON\ VS.\ DEX}$=0.0013, **$P_{DEX\ VS.\ DEX\ +\ NaClO3}$ =0.0083, one-way ANOVA with Tukey's multiple comparisons test). **(C)** Quantification of ELISA for total Tau levels in EV-depleted media from the indicated conditions, with values normalized to CON condition (***$P_{CON\ VS.\ DEX}$=0.0002, **$P_{DEX\ VS.\ DEX\ +\ m\text{-}\beta\text{-}c}$ =0.0024, one-way ANOVA with Tukey's multiple comparisons test). **(D)** Quantification of ELISA for total Tau levels in EV-depleted ACSF from the indicated conditions, with values normalized to CON condition (**$P_{CON\ VS.\ DEX}$=0.0027, *$P_{DEX\ VS.\ DEX\ +\ +\ m\text{-}\beta\text{-}c}$ =0.0237, one-way ANOVA with Tukey's multiple comparisons test). (**E, F**) Quantification of AT8 or PHF1 levels in EV-depleted media from hippocampal neurons treated with $NaClO_3$, expressed as ratio of total Tau (see Fig. **2A-D**) (ns, one-way ANOVA with Tukey's multiple comparisons test). (**G, H**) Quantification of AT8 or PHF1 levels in EV-depleted ACSF from indicated conditions, expressed as ratio of total Tau (see Fig. **2E-H**) (ns, one-way ANOVA with Tukey's multiple comparisons test; n=3 samples/condition). (**I, J**) Quantification of AT8 or PHF1 levels in EV-depleted media from hippocampal neurons treated with m-β-c, expressed as ratio of total Tau (see Fig. **2I-L**) (ns, one-way ANOVA with Tukey's multiple comparisons test). (**K, L**) Quantification of AT8 or PHF1 levels in EV-depleted ACSF from indicated conditions, expressed as ratio of total Tau (see Fig. **2M-P**) (ns, one-way ANOVA with Tukey's multiple comparisons test). (**M**) Quantification of total Tau (ELISA), LDH, and ATP levels in EV-depleted media from primary neurons treated as indicated, with values normalized to CON condition ($P_{CON\ VS.\ 2\text{-}DG}$=0.7746 for Tau ELISA assay, $P_{CON\ VS.\ 2\text{-}DG}$=0.5949 for LDH assay, ****$P_{CON\ VS.\ 2\text{-}DG}$<0.0001 for ATP assay, unpaired two-tailed t-test). (**N, O**) Representative immunoblots and quantification of PHF1 levels in lysates from hippocampal neurons treated as indicated, expressed as ratio of total Tau (*$P_{CON\ VS.\ DEX}$ =0.0298, $P_{CON\ VS.\ DEX\ +\ TDZD}$ =0.5748, $P_{CON\ VS.\ DEX\ +\ TTX}$ =0.0754, one-way ANOVA with Tukey multiple comparisons test). (**P**) Quantification of body weight loss in mice treated for 21 days with CON, DEX, DEX + MIF, or DEX + EGCG ($P_{CON\ VS.\ DEX,\ DEX+MIF,\ DEX+EGCG}$<0.001 (days 21), **$P_{CON\ VS.\ DEX}$ <0.01 (days 15-20), $^{\#}P_{CON\ VS.\ DEX+MIF,\ DEX+EGCG}$<0.05 (days 12-14), 2-way ANOVA with Tukey multiple comparisons test; n=3-7 mice/condition). Data is presented as mean ± SEM with n= 3-4 samples/group (results were confirmed in two independent experiments).

Figure S3. Lower magnification images of Tau spreading *in vivo* and demonstration that EGCG inhibits Tau secretion and spreading. (A-D) Representative images showing the colocalization of hTau (far red) and GFP (green) in hippocampal area CA1 neurons of mice treated as indicated. Nuclei are stained with DAPI (blue). Right columns show enlarged regions (indicated by yellow boxes). Scale bars, 500 μm. (**E-H**) Representative immunoblots (**E**) and quantification **(F-H)** of AT8, PHF1 and Tau immunoreactivity in ACSF from brain slices perfused with vehicle (CON), DEX, or DEX + EGCG. Intensity values are normalized to CON condition (***$P_{\text{CON VS. DEX}}$ =0.0003, **$P_{\text{DEX VS. DEX + EGCG}}$ =0.0042 for **F**, *$P_{\text{CON VS. DEX}}$ =0.0124, *$P_{\text{DEX VS. DEX + EGCG}}$=0.0138 for **G**, **$P_{\text{CON VS. DEX}}$ =0.0052, *$P_{\text{DEX VS. DEX + EGCG}}$=0.0115 for **H**, one-way ANOVA with Tukey multiple comparisons test; n=3 samples/condition). **(I)** Quantification of ELISA for total Tau levels in EV-depleted ACSF from the indicated conditions, with values normalized to CON condition (**$P_{\text{CON VS. DEX}}$=0.0012, **$P_{\text{DEX VS. DEX + EGCG}}$ =0.0014, one-way ANOVA with Tukey multiple comparisons test; n=3 samples/condition).

Figure S4. Immunoblots. The full immunoblots used in each figure are shown here, with blue boxes indicating the regions shown.

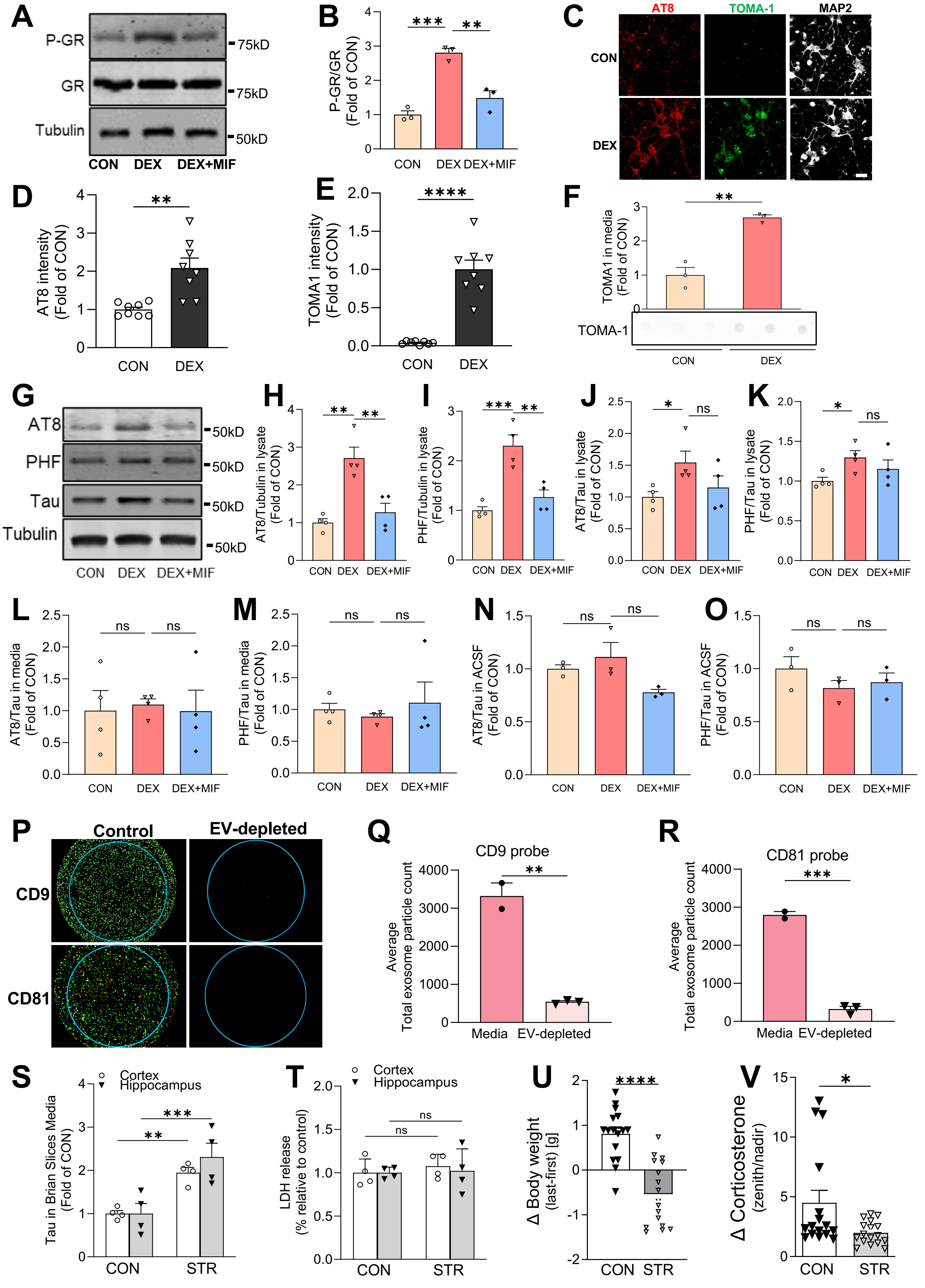
A
P-GR
GR
Tubulin
75kD
75kD
50kD
CON DEX DEX+MIF
B
P-GR/GR (Fold of CON)
CON DEX DEX+MIF
C
AT8
TOMA-1
MAP2
CON
DEX
D
AT8 intensity (Fold of CON)
CON DEX
E
TOMA1 intensity (Fold of CON)
CON DEX
F
TOMA1 in media (Fold of CON)
TOMA-1
CON DEX
G
AT8
PHF
Tau
Tubulin
50kD
CON DEX DEX+MIF
H
AT8/Tubulin in lysate (Fold of CON)
I
PHF/Tubulin in lysate (Fold of CON)
J
AT8/Tau in lysate (Fold of CON)
K
PHF/Tau in lysate (Fold of CON)
L
AT8/Tau in media (Fold of CON)
M
PHF/Tau in media (Fold of CON)
N
AT8/Tau in ACSF (Fold of CON)
O
PHF/Tau in ACSF (Fold of CON)
ns
P
Control
EV-depleted
CD9
CD81
Q
CD9 probe
Average Total exosome particle count
Media EV-depleted
R
CD81 probe
S
Cortex
Hippocampus
Tau in Brian Slices Media (Fold of CON)
CON STR
T
LDH release (% relative to control)
U
Δ Body weight (last-first) [g]
V
Δ Corticosterone (zenith/nadir)

Figure S1

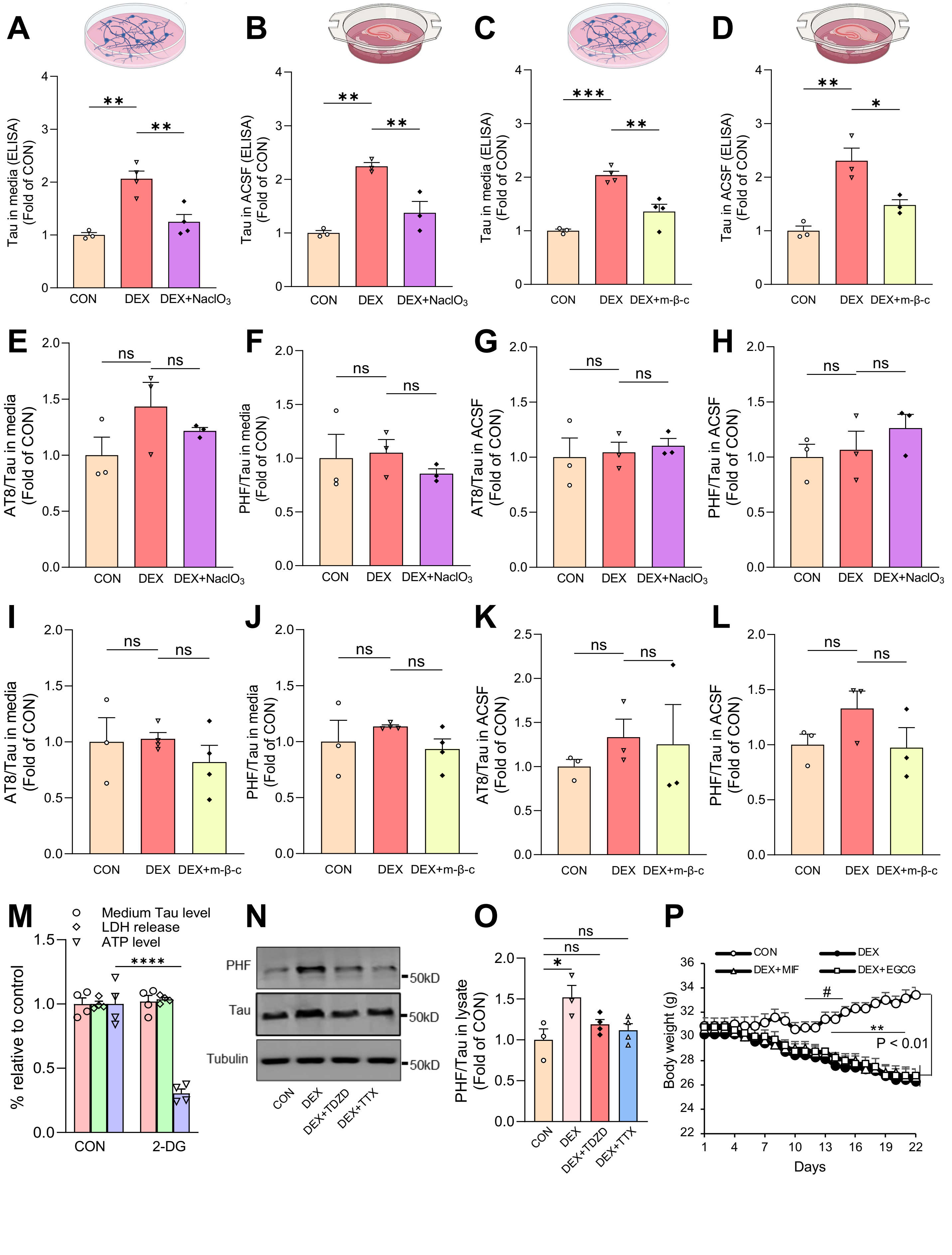

A
Tau in media (ELISA) (Fold of CON)
CON
DEX
DEX+NaclO3
**
B
Tau in ACSF (ELISA) (Fold of CON)
C
D
DEX+m-β-c
***
*
E
AT8/Tau in media (Fold of CON)
ns
F
PHF/Tau in media (Fold of CON)
G
AT8/Tau in ACSF (Fold of CON)
H
PHF/Tau in ACSF (Fold of CON)
I
J
K
L
M
Medium Tau level
LDH release
ATP level
% relative to control
2-DG
****
N
PHF
Tau
Tubulin
50kD
DEX+TDZD
DEX+TTX
O
PHF/Tau in lysate (Fold of CON)
P
DEX+MIF
DEX+EGCG
Body weight (g)
Days
#
P < 0.01

Figure S2

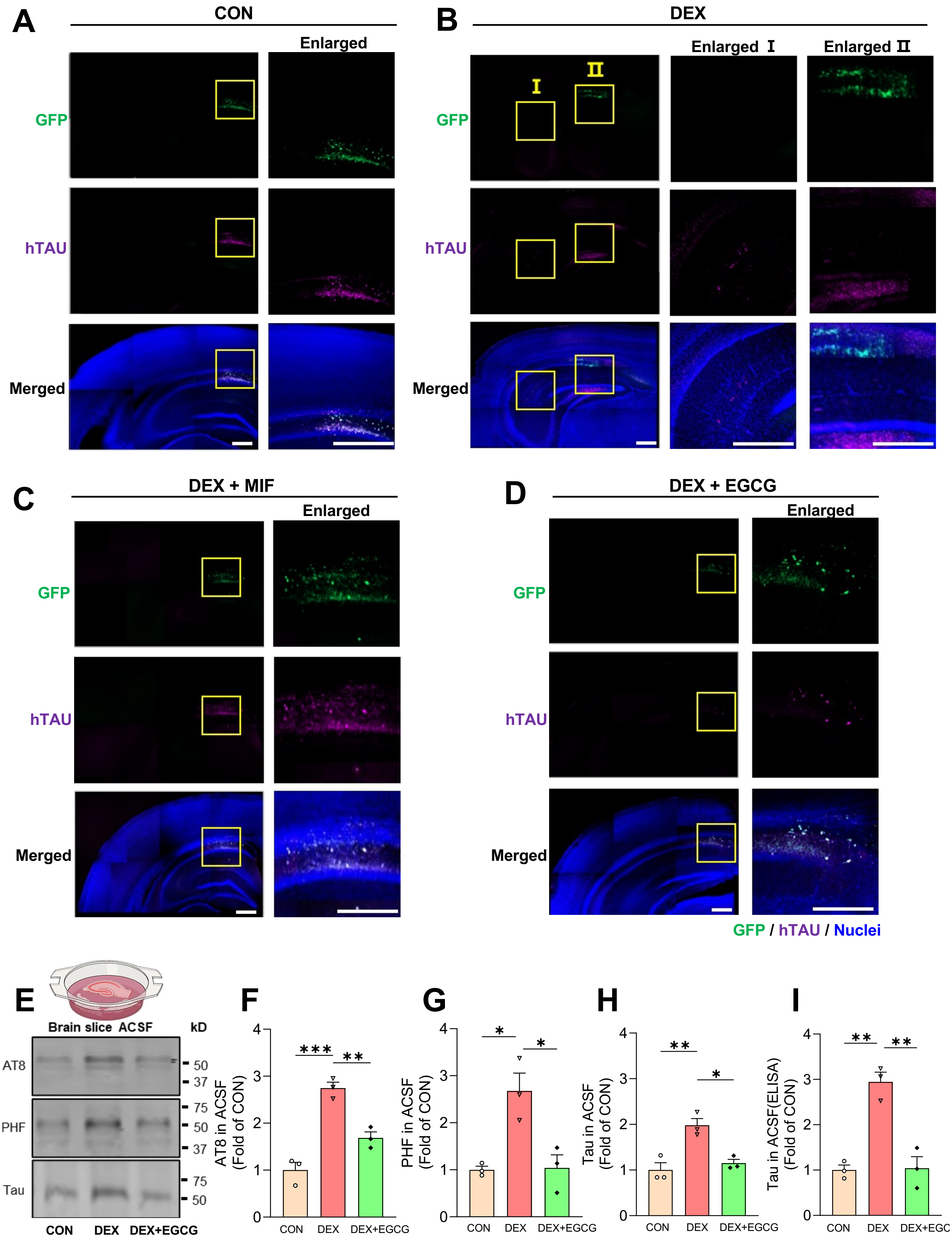
A
CON
Enlarged
GFP
hTAU
Merged
B
DEX
Enlarged I
Enlarged II
I
II
C
DEX + MIF
D
DEX + EGCG
GFP / hTAU / Nuclei
E
Brain slice ACSF
kD
AT8
PHF
Tau
50
37
75
CON
DEX
DEX+EGCG
F
AT8 in ACSF (Fold of CON)
G
PHF in ACSF (Fold of CON)
H
Tau in ACSF (Fold of CON)
I
Tau in ACSF(ELISA) (Fold of CON)

Figure S3

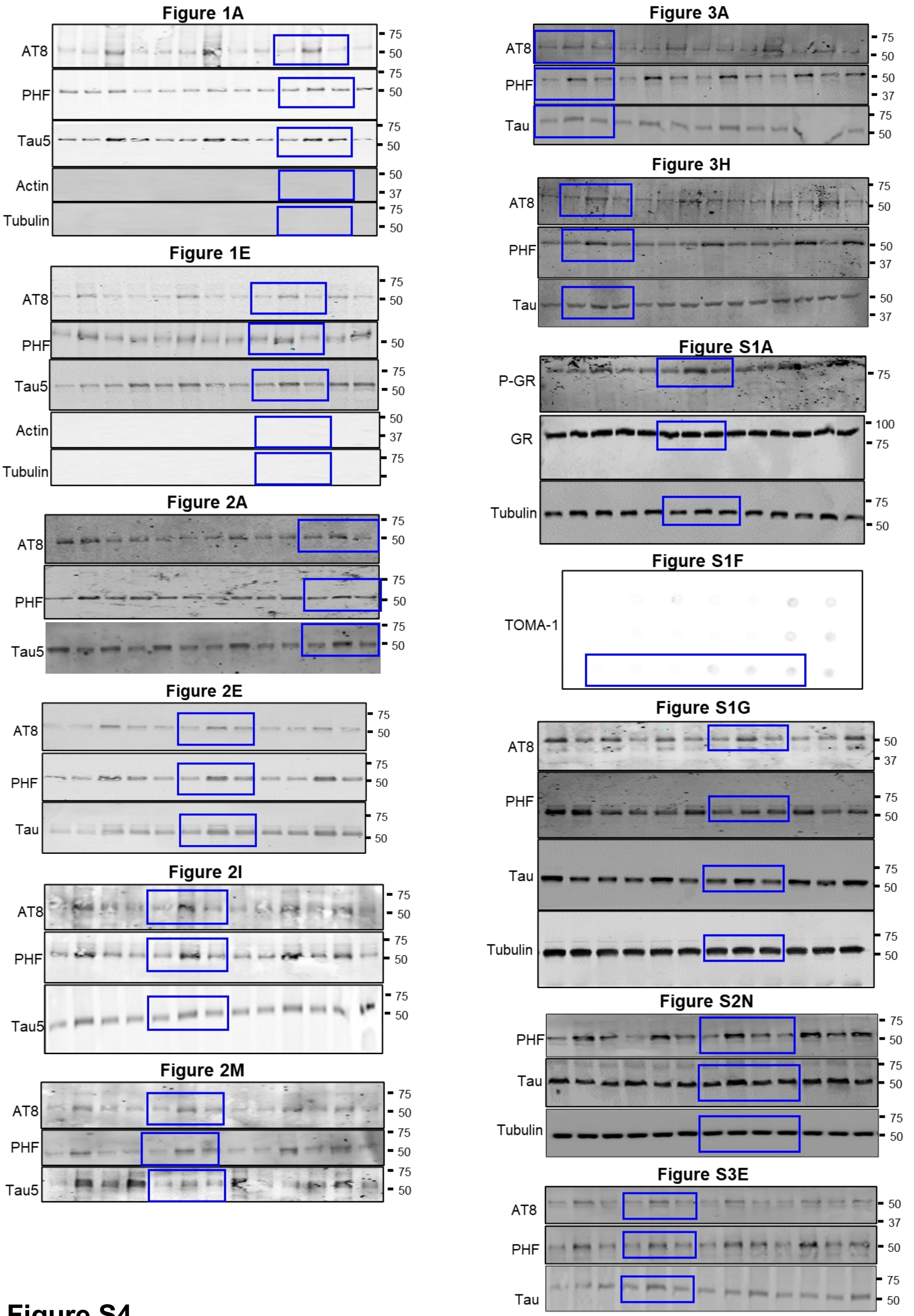
Figure 1A
AT8
PHF
Tau5
Actin
Tubulin
75
50
37
Figure 1E
AT8
PHF
Tau5
Actin
Tubulin
Figure 2A
AT8
PHF
Tau5
Figure 2E
AT8
PHF
Tau
Figure 2I
AT8
PHF
Tau5
Figure 2M
AT8
PHF
Tau5
Figure 3A
AT8
PHF
Tau
Figure 3H
AT8
PHF
Tau
Figure S1A
P-GR
GR
Tubulin
100
Figure S1F
TOMA-1
Figure S1G
AT8
PHF
Tau
Tubulin
Figure S2N
PHF
Tau
Tubulin
Figure S3E
AT8
PHF
Tau

Figure S4
